# Complex‐Amplitude Programmable Versatile Metasurface Platform Driven by Guided Wave

**DOI:** 10.1002/advs.202309873

**Published:** 2024-03-14

**Authors:** Jian‐Qiao Han, Fan‐Yi Meng, Chunsheng Guan, Cong Wang, Tao Jin, Tong Cai, Chang Ding, Shah Nawaz Burokur, Qun Wu, Xumin Ding

**Affiliations:** ^1^ Department of Microwave Engineering Harbin Institute of Technology Harbin 150001 P. R. China; ^2^ Air and Missile Defense College Air Force Engineering University Xi'an 710051 P. R. China; ^3^ Air Force Engineering University Xi'an 710051 P. R. China; ^4^ LEME, UPL Univ Paris Nanterre Ville d'Avray F92410 France; ^5^ Advanced Microscopy and Instrumentation Research Center Harbin Institute of Technology Harbin 150080 P. R. China; ^6^ Key Laboratory of Millimeter Waves Nanjing 210096 P. R. China

**Keywords:** ariy beam, complex‐amplitude modulation, guided wave‐driven metasurface, low sidelobe‐level beam scanning

## Abstract

Metasurfaces have shown unparalleled controllability of electromagnetic (EM) waves. However, most of the metasurfaces need external spatial feeding sources, which renders practical implementation quite challenging. Here, a low‐profile programmable metasurface with 0.05λ_0_ thickness driven by guided waves is proposed to achieve dynamic control of both amplitude and phase simultaneously. The metasurface is fed by a guided wave traveling in a substrate‐integrated waveguide, avoiding external spatial sources and complex power divider networks. By manipulating the state of the p‐i‐n diodes embedded in each meta‐atom, the proposed metasurface enables 1‐bit amplitude switching between radiating and nonradiating states, as well as a 1‐bit phase switching between 0° and 180°. As a proof of concept, two advanced functionalities, namely, low sidelobe‐level beam scanning and Airy beam generation, are experimentally demonstrated with a single platform operating in the far‐ and near‐field respectively. Such complex‐amplitude, programmable, and low‐profile metasurfaces can overcome integration limitations of traditional metasurfaces, and open up new avenues for more accurate and advanced EM wave control within an unprecedented degree of freedom.

## Graphical abstract

A low‐profile programmable metasurface with 0.05λ_0_ thickness driven by guided waves is proposed to achieve dynamic control of both amplitude and phase simultaneously. As a proof of concept, two advanced functionalities, namely, low sidelobe‐level beam scanning and Airy beam generation, are experimentally demonstrated with a single platform operating in the far‐ and near‐field respectively.

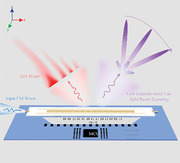

## 1 Introduction

Metasurfaces,^[^ ^1^, ^2^ ^]^ the 2D version of metamaterials, possess the advantage of superior ability in wavefront tailoring. By changing the resonance characteristics,^[^ ^3^, ^4^ ^]^ transmission characteristics,^[^ ^5^, ^6^, ^7^ ^]^ rotation symmetry,^[^ ^8^, ^9^ ^]^ and nonlinear feature^[^ ^10^, ^11^ ^]^ of the meta‐atom, the amplitude,^[^ ^12^, ^13^ ^]^ phase,^[^ ^14^, ^15^ ^]^ polarization,^[^ ^16^, ^17^ ^]^ and frequency^[^ ^18^, ^19^ ^]^ of EM waves can be manipulated. Various applications have been demonstrated such as beam scanning,^[^ ^20^, ^21^ ^]^ multi‐beam generation,^[^ ^22^, ^23^ ^]^ polarization transformation,^[^ ^24^, ^25^ ^]^ radar cross‐section reduction,^[^ ^26^, ^27^ ^]^ Airy beam generation,^[^ ^28^, ^29^, ^30^, ^31^ ^]^ image processing,^[^ ^32^, ^33^ ^]^ and focusing.^[^ ^34^, ^35^ ^]^ However, most metasurfaces require external spatial feeding sources, increasing their profiles.^[^ ^36^ ^]^ Although folded‐reflection,^[^ ^37^ ^]^ folded‐transmission,^[^ ^38^ ^]^ and Fabry–Perot^[^ ^39^ ^]^ type metasurfaces have been proposed to reduce the distance between the feeding source and the metasurface, the air gap between the metasurface and feeding source still hinders them from real‐world integrated applications. To further improve the integration level, radiation‐type metasurfaces^[^ ^40^, ^41^, ^42^ ^]^ and guided wave‐driven metasurfaces^[^ ^43^, ^44^, ^45^, ^46^, ^47^ ^]^ have received extensive attention. Radiation‐type metasurfaces can be seen as antenna arrays, which employ Wilkinson power dividers as feeding sources for each meta‐atom.^[^ ^40^, ^48^ ^]^ Such a metasurface enables the capability to manipulate arbitrary phases, linear polarization states, and energy distribution simultaneously. They are not only based on geometric and resonant phases of the meta‐atom,^[^ ^40^, ^41^, ^48^ ^]^ but also provide additional initial phase shift and amplitude distribution through the power dividers.^[^ ^42^, ^49^ ^]^ However, as the scale of radiation‐type metasurface increases, the design of the power divider network becomes extremely complex, and the losses introduced by the microstrip lines cannot be neglected.

Guided wave‐driven metasurfaces utilize meta‐atoms as couplers to transform the guided wave into a spatial wave.^[^ ^50^, ^51^ ^]^ Since the feeding network for each meta‐atom is avoided, it is easier to achieve large‐scale^[^ ^52^ ^]^ and conformal apertures^[^ ^53^ ^]^ compared to radiation‐type metasurface. By manipulating the amplitude.^[^ ^43^, ^54^ ^]^ phase^[^ ^55^, ^56^ ^]^ and impedance^[^ ^52^, ^57^ ^]^ of the meta‐atoms, guided wave‐driven metasurfaces have been demonstrated to achieve various functionalities, such as focusing,^[^ ^25^, ^58^ ^]^ beam shaping,^[^ ^59^ ^]^ Airy beam^[^ ^45^ ^]^ and vortex beam generation.^[^ ^60^ ^]^ Dynamic complex‐amplitude modulation can achieve more advanced wavefront tailoring, such as high‐quality holograms and beamforming,^[^ ^61^, ^62^, ^63^, ^64^, ^65^ ^]^ while reported guided wave‐driven metasurfaces have been unable to achieve such performances so far. In the next generation of wireless systems, cognitive sensing, and imaging devices, high‐quality wavefront regulation with low‐profile and minimalist feeding network will be vital and challenging, which cannot be achieved by only‐amplitude or only‐phase metasurfaces, thereby necessitating both amplitude and phase modulation schemes.

Herein, we propose a guided wave‐driven metasurface for dynamic and simultaneous control of both amplitude and phase. In order to avoid external spatial sources and complex power divider networks, the metasurface is fed by the guided wave traveling in a  substrate‐integrated waveguide (SIW) structure. Two p‐i‐n diodes are loaded in each meta‐atom to realize 1‐bit amplitude switching between radiation and nonradiation states, as well as 1‐bit phase switching between 0° and 180°. Combined with the genetic algorithm (GA), a low sidelobe‐level (SLL) reconfigurable microwave antenna is demonstrated, which can achieve beam scanning in the far‐field region from ─45° to +45° with an SLL less than ─14 dB. In addition, high‐quality Airy beams are generated utilizing the same platform operating in the near‐field region, with dynamic control of the scale level and steering angle of the latter Airy beams. Measurements performed on a fabricated prototype show that experimental results agree well with the simulated and theoretical ones, thereby validating the feasibility of the proposed design.

## 2 Guided Wave‐Driven Metasurface Design

**Figure 1:**
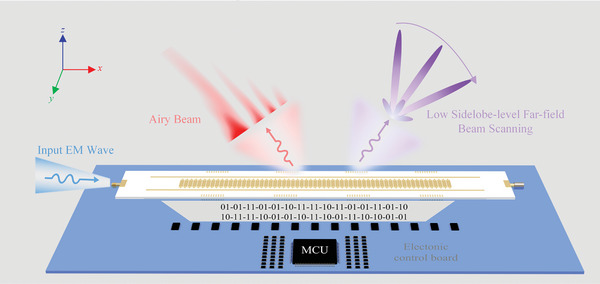
Conceptual illustration of the CAP metasurface. The designed guided wave‐driven CAP metasurface consists of 61 meta‐atoms, with a period of 4.8 mm. By loading p‐i‐n diodes in the meta‐atom, the element can achieve 1‐bit amplitude switching between radiating ("10" or "01") and nonradiating states ("11"), as well as 1‐bit phase switching between 0° ("01") and 180° ("10"). In this concept, the CAP metasurface driven by guided wave achieves low‐side lobe‐level beam scanning and Airy beam generation.

The conceptual illustration of a complex‐amplitude programmable (CAP) metasurface driven by guided wave is shown in **Figure** **1**. The designed metasurface implementing complex‐amplitude modulation can couple guided wave traveling in a SIW structure into free space. The p‐i‐n diodes loaded in the CAP metasurface are controlled by a microcontroller unit (MCU), enabling dynamic programming of the radiation states of the meta‐atoms. The geometric structural diagram of the metasurface can be viewed in Text S1 (Supporting Information).

**Figure 2:**
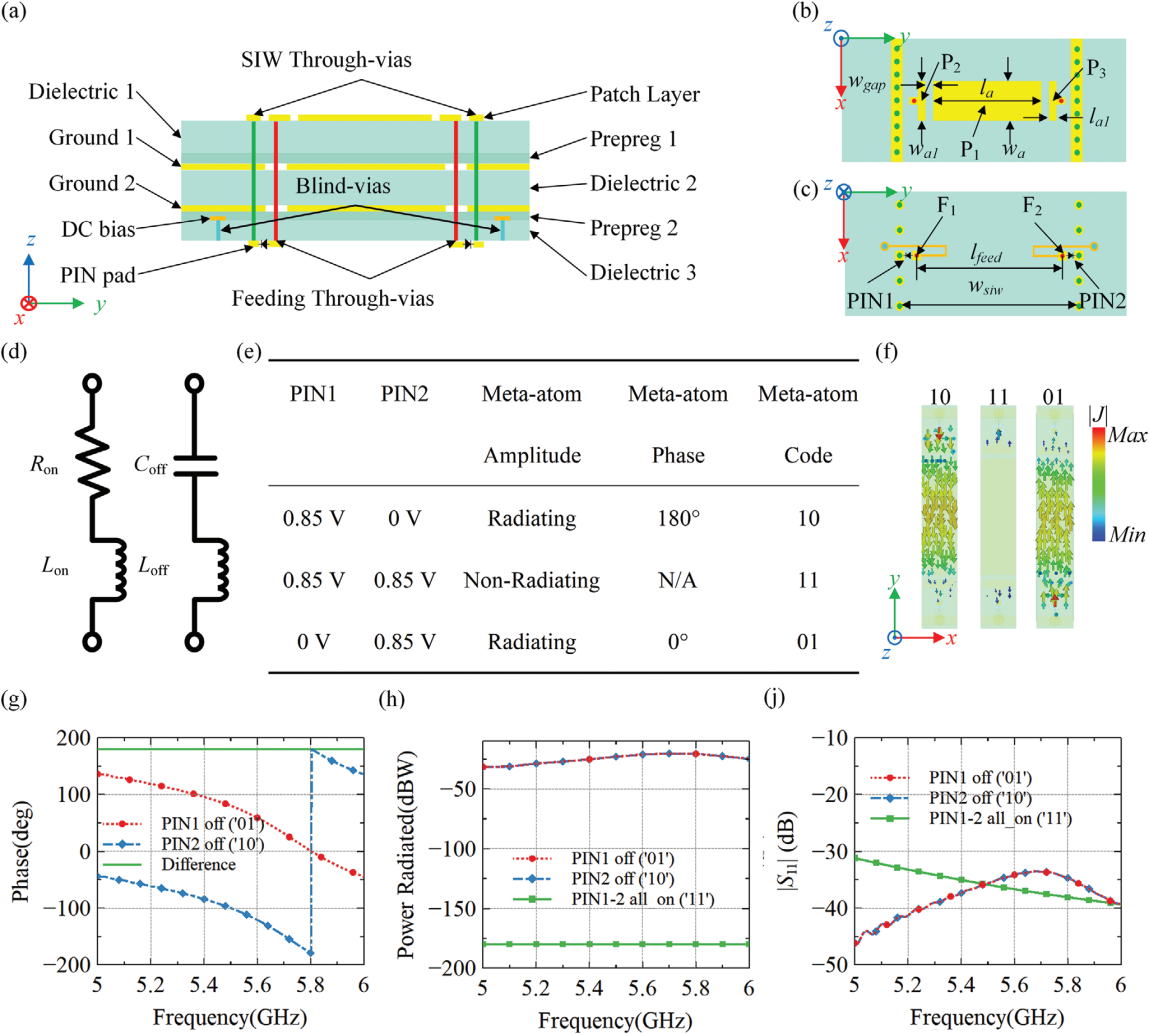
Geometry and radiation characteristics of the elementary meta‐atom of the CAP metasurface driven by a guided wave. a) Side view of the meta‐atom. b) Top view of the meta‐atom. c) Bottom view of the meta‐atom. d) Equivalent circuit of the p‐i‐n diode (SMP1321‐079LF, Skyworks).^[^ ^69^ ^]^ When the p‐i‐n diode state is in the "on" state, the simplified equivalent circuit is a resistance *R*_on_ = 2.2 Ω in series with an inductance *L*_on_ = 0.7 nH. For the "off" state of the p‐i‐n diode, the simplified equivalent circuit is a capacitance *C*_off_ = 0.32 pF in series with an inductance *L*_on_ = 0.7 nH. e) Operating mechanism of the meta‐atom for different electrical states. f) Surface current distributions of the meta‐atom when PIN_1_ state is "on" and PIN_2_ state is "off" ("10"), PIN_1_ and PIN_2_ states are "on" ("11"), and PIN_1_ state is "off" and PIN_2_ state is "on" ("01"). g) Simulated radiation phase of the meta‐atom. h) Simulated radiation energy of the meta‐atom. i) Simulated reflection coefficient of the meta‐atom. The fixed geometrical parameters are: *w*_a_ = 4.0 mm, *l*_a_ = 17.0 mm, *w*_gap_ = 0.4 mm, *w*_a2_ = 1.0 mm, *l*_feed_ = 23.0 mm, and *w*_siw_ = 26.0 mm.

The geometry of the elementary meta‐atom of the CAP metasurface is shown in **Figure** **2**a–c. The meta‐atom consists of three dielectric substrates (Dielectric 1–3), two bonding layers (Prepreg 1–2), and five metal layers (Patch layer, Ground 1, Ground 2, DC bias, and PIN pad), as shown in Figure 2a. Dielectrics 1, 2, and 3 are made of F4BME220 (*ε_r_* = 2.2, tan *δ* = 0.001), F4BME300 (*ε_r_* = 3.0, tan *δ* = 0.0018), and F4BME220 (*ε_r_* = 2.2, tan *δ* = 0.001), respectively, with thickness 1.50, 1.00, and 0.25 mm. The dielectric bonding material is Rogers 4450F (*ε_r_* = 3.52, tan *δ* = 0.004) with a thickness of 0.2 mm. Grounds 1 and 2, located on the top and bottom faces of Dielectric 2, together with the metal through vias form a SIW structure that drives the CAP metasurface. The DC bias is divided into two parts located on the top and bottom faces of Dielectric 3, which are connected using metal blind vias. PIN pads for soldering p‐i‐n diodes that control the amplitude and phase of the meta‐atom are also located on the bottom face of Dielectric 3, as shown in Figure 2c. The influence of the DC bias network on the meta‐atom characteristics is given in Text S2 (Supporting Information). The meta‐atom comprises a rectangular metal patch (P_1_), two ‘T’ shaped metal patches (P_2_ and P_3_), and feeding through vias (F_1_ and F_2_), as shown in Figure 2b. Two p‐i‐n diodes (PIN_1_ and PIN_2_), whose equivalent electric circuit is shown in Figure 2d, are placed on the lower face of Dielectric 3. The positive terminals of the p‐i‐n diodes are connected to F_1_ and F_2_, while the negative terminals are connected to the SIW through vias, as depicted in Figure 2a. The other ends of F_1_ and F_2_ are connected to P_2_ and P_3_, respectively.

The designed meta‐atom has three operating states, as shown in Figure 2e. When both PIN_1_ and PIN_2_ states are ″on″ or ″off″ the meta‐atom is in the nonradiating state (expressed as ″11″ or ″00″ the functions implemented by ″11″ and ″00″ are the same, and both are represented by ″11″ in the following text.). When one p‐i‐n diode state is ″off″ and the other is ″on″ the meta‐atom is excited, and for different ″off″ p‐i‐n diode, the two radiation phase responses of 0° (expressed as ″01″) or 180° (expressed as ″10″) can be realized. Figure 2f shows the simulated results of the meta‐atom surface currents distribution at 5.4 GHz for the three operating states. The surface currents of the meta‐atom are inverted when the off‐states of PIN_1_ and PIN_2_ are switched. Thus, the designed meta‐atom has a stable 180° phase difference. When both PIN_1_ and PIN_2_ states are ″on″, there is almost no surface current on the surface of the meta‐atom, which indicates that the meta‐atom is not excited. In Figure 2g, the simulation results of the radiation phase of the meta‐atom can achieve a 180° phase difference in the 5–6 GHz frequency range when it is set to different operating states. Figure 2h displays the simulation results of the radiated energy of the meta‐atom when it is excited and not excited. It is worth noting that the radiated energy remains the same when the meta‐atom is excited for two‐phase states. The difference in radiated energy between the excited and nonexcited states can reach up to 140 dB. Conversely, when the meta‐atom is not excited, there is almost no energy radiated into free space. Figure 2i shows the simulation results of *S*_11_ of the three states, and the reflection coefficient is less than −33 dB in the frequency range of 5–6 GHz. The lower *S*_11_ level of the meta‐atom can effectively suppress the open band phenomenon in guided wave‐driven metasurfaces,^[^ ^66^ ^]^ similar to the mechanism of the open band phenomenon in leaky wave antennas.^[^ ^67^, ^68^ ^]^

## 3 Results and Analyses

### 3.1 Far‐Field Low Sidelobe‐Level Beam Scanning

When the angle of the radiated beam is designed to be *θ*_0_, the phase response *φ*_n_ of the *n*‐th meta‐atom of the CAP metasurface (as shown in Figure S3, Supporting Information) can be represented as follows^[^ ^70^ ^]^

(1) $$\varphi_{n}=n\times d\times \left(k_{s}-k_{0}\sin\theta_{0}\right)+\varphi_{0},n=1,2,3,4,\text{…},61$$

where *k*_0_ is the wavenumber in free space, *k*_s_ is the wavenumber in the SIW structure, *d* is the distance between adjacent elements and *φ*_0_ is the initial phase. More details of the principle of far‐field radiated beam scanning are provided in Text S3 (Supporting Information). As the p‐i‐n diode has only two operating states (on‐state and off‐state), the p‐i‐n diode loaded metasurface can only achieve quantized phase levels^[^ ^71^, ^72^ ^]^. In our proposed meta‐atom, only 0 and *π* phase changes can be obtained by controlling the operating states of the loaded p‐i‐n diodes. Thus, the calculated continuous phase distribution from Equation (1) should be quantized into two‐phase values of 0 and *π*. The quantization phase response *φ*_qua,n_ of the *n*‐th meta‐atom can be represented as

(2) $$\varphi_{\mathrm{qua},n}=\left\{\begin{array}{c} 0,\mathrm{if}0\leq \varphi_{\mathrm{req},n}<\pi \\ \pi ,\mathrm{if}\pi \leq \varphi_{\mathrm{req},n}\leq 2\pi \end{array}\right.$$

The proposed design can be scaled up to multi‐bit phase reconfigurability by increasing the number of p‐i‐n diodes in the meta‐atoms. However, considering the design complexity, insertion losses, and manufacturing costs increased by the number of p‐i‐n diodes, 1‐bit switching is utilized here as a good tradeoff.

Thus, the array factor can be expressed as

(3) $$f\left(\theta \right)=\sum_{n=1}^{61}A_{n}e^{-j(nd\left(k_{0}\sin\theta -k_{s}\right)-\varphi_{\mathrm{qua},n})},n=1,2,3,4,\text{…},61$$

where *A*_n_ is the amplitude of the *n*‐th meta‐atom.

Due to the 1‐bit phase modulation, some undesired higher harmonics will be introduced. The excited higher harmonics give rise to high SLLs if the angular frequency is within the range of ‐*k*_0_ to *k*_0_. Different from higher‐order diffraction related to the periodicity in phase gradient metasurfaces, higher harmonics here refer to undesired waves caused by the phase quantization error.^[^ ^73^ ^]^ To suppress these higher harmonics, the spatial spectrum of the far‐field pattern of the 1‐bit phase modulation metasurface is analyzed. The far‐field pattern calculated using the Fourier transform can be represented as^[^ ^66^, ^74^ ^]^

(4) f(θ)=Cbm∑a=−∞∞∑m=1∞sinβx−βa,m,−+jαLL22βx−βa,m,−+jα+sinβx−βa,m,++jαLL22βx−βa,m,++jα

where *β*_*x*_ = *k*_0_sin θ, *C* is the coefficient after Fourier transform, *α* is the attenuation constant and *L* is the length of the metasurface. *b*_m_ is the coefficient generated by the Fourier transform of the 1‐bit phase modulation function, expressed as

(5) $$b_{m}=\left\{\begin{array}{cc} \frac{4}{m\pi } & ,m\;\mathrm{is}\;\mathrm{odd}\\ 0 & ,m\;\mathrm{is}\;\mathrm{even} \end{array}\right.$$

The expression for *β*_a,m,‐_ and *β*_a,m,+_ can be represented as

(6) $$\begin{array}{c} \beta_{a,m,-}=k_{s}+2a\pi /d-2m\pi /T_{1}\\ \beta_{a,m,+}=k_{s}+2a\pi /d+2m\pi /T_{1} \end{array}$$

where *T*_1_ is the modulation period of the 1‐bit phase modulation function, *T*_1_ = 2*π* / (*k*_s_–*k*_0_sin*θ*_0_).

**Figure 3:**
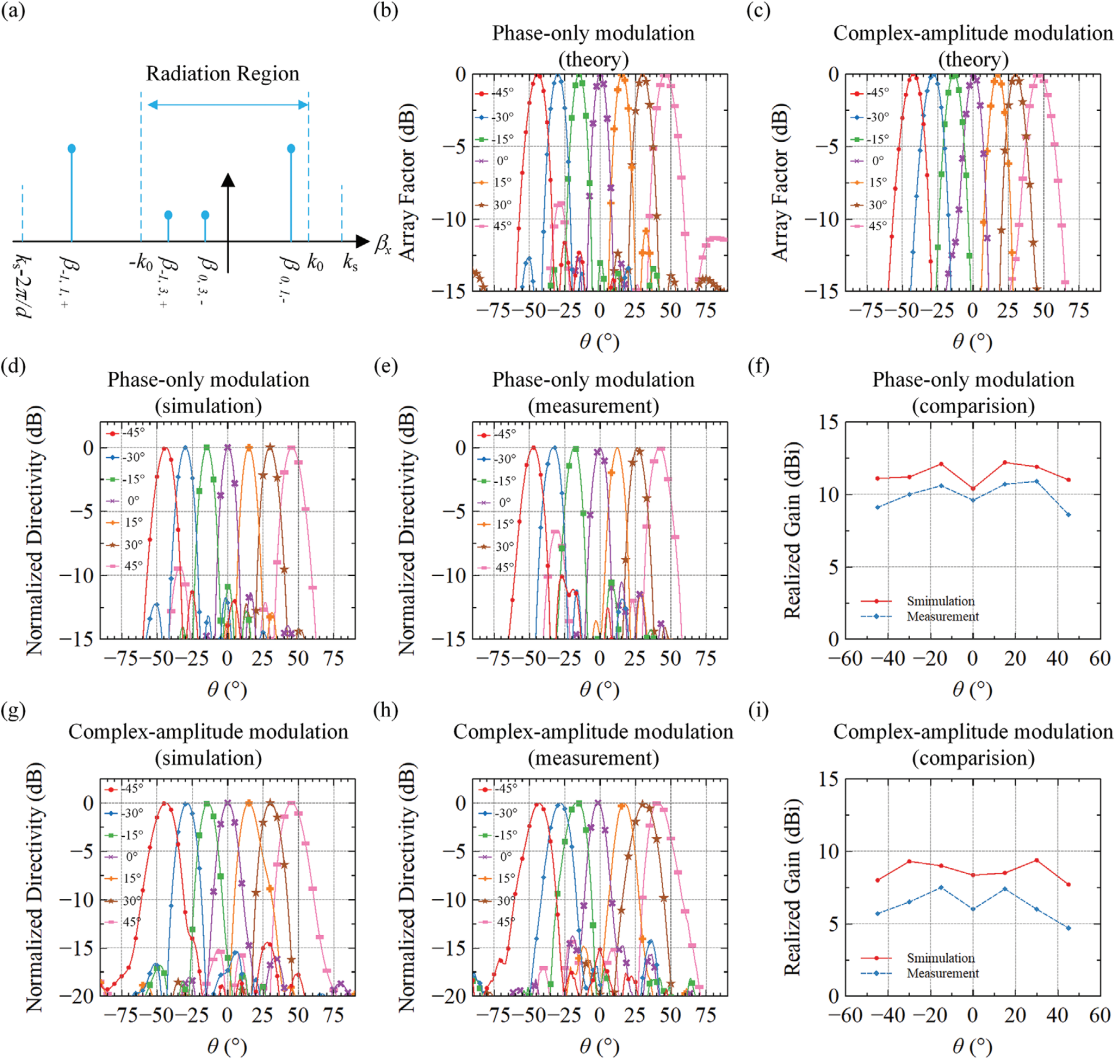
Beam scanning by guided wave‐driven metasurface. a) 1‐bit phase modulated spatial spectrum. b) Theoretical far‐field patterns for 1‐bit phase modulation. c) Theoretical far‐field patterns for complex‐amplitude modulation. d–f) Results for 1‐bit phase modulation at 5.4 GHz: d) Simulations, e) Measurements, f) Realized gain. g–i) Results for complex amplitude modulation at 5.4 GHz: g) Simulations, h) Measurements, i) Realized gain.

According to Equations (4)–(6), the spatial spectrum of the guided wave‐driven metasurface can be calculated and is depicted in **Figure** **3**a. When the higher harmonics *β*_0,3,‐_ (*β*_0,3,‐_ = 3*k*_0_sin*θ*_0_ – 2*k*_s_) and *β*_−1,3,+_ (*β*_−1,3,+_ = 4*k*_s_ – 3*k*_0_sin*θ*_0_ – 2*π*/*d*) are located in the radiation region, they can radiate into free space and lead to high SLL. When the spacing *d* between adjacent meta‐atoms decreases to a certain value, the higher harmonic *β*_−1,3,+_ can be moved out of the radiation region. The condition under which the higher harmonic *β*_−1,3,+_ is suppressed can be expressed as

(7) maxβ−1,3,+=4ks−3k0sinθ0−2π2πdd<−k0

For *θ*_0_ = ‐*π*/2, *β*_−1,3,+_ reaches the maximum value. Therefore, when *d* < *π /* (2*k*_0_ + 2*k_s_*), the higher harmonic *β*_−1,3,+_ is suppressed. Since the high harmonic *β*_0,3,‐_ is independent of *d*, the condition for suppressing *β*_0,3,‐_ is *k*_s_ > 2*k*_0_. However, it is very difficult to design a SIW structure that meets the above criteria. When *k*_0_ < *k*_s_ < 2*k*_0_, *β*_0,3,‐_ is suppressed in −*π*/2 < *θ*_0_ < sin^−1^[(2*k*_s_–*k*_0_)/3*k*_0_]. In sin^−1^[(2*k*_s_ – *k*_0_)/3*k*_0_] ≤ *θ*_0_ < *π*/2, the pointing angle *θ*_0,3,‐_ of the high SLL caused by *β*_0,3,‐_ can be expressed as

(8) $$\theta_{0,3,-}=\sin^{-1}\left(3\sin\theta_{0}-\frac{2k_{s}}{k_{0}}\right)$$

The wavenumber *k*_s_ of the designed SIW structure operating at 5.4 GHz is 147.0 rad m.^−1^ According to Equation (7), it can be inferred that when the distance between adjacent elements *d* is less than 6.0 mm, higher harmonic *β*_−1,3,+_ will be suppressed. Consequently, in this design, the distance between adjacent elements *d* considered is set at 4.8 mm to ensure the effective suppression of higher harmonic *β*_−1,3,+_. In such cases, the predicted radiation patterns are shown in Figure 3b (The codes of the meta‐atoms on the CAP metasurface for different radiated beam scanning angles are shown in Figure S5a, Supporting Information). When the radiated beam scanning angle is 45°, the SLL at *θ* = −28.5° is −8.8 dB, and according to Equation (8), it can be seen that at this time, *θ*_0,3,‐_ = −28.56°, which verifies the sidelobe is generated by the excitation of the higher harmonic *β*_0,3,‐_. However, the SLL for other radiated beam scanning angles is maintained lower than −10 dB. Therefore, it is demonstrated that reducing the element spacing can suppress the radiation of higher harmonic *β*_−1,3,+_ commonly presented in the 1‐bit phase modulation function.

The amplitude of the proposed meta‐atom has two states: ″radiating″ (″10″ or ″01″) and ″nonradiating″ (″11″). By utilizing theGA to optimize the amplitude of the meta‐atom, the array is transformed from a uniform linear array to a sparse linear array. The incorporation of amplitude modulation can disrupt the periodic nature of 1‐bit phase modulation, effectively reducing the generation of higher harmonics caused by the 1‐bit phase modulation function. The first and end meta‐atoms remain in the ″radiating″ state to ensure that the array aperture keeps unchanged. More details of the GA are provided in Text S4 (Supporting Information). It can be observed that when the number of nonradiating elements is different, the final optimized sidelobe level is also different. The more the number of nonradiating components, the lower the SLL. Therefore, in the following simulation experiment, the number of nonradiating elements is selected as 21. For different radiated beam scanning angles, their corresponding predicted far‐field patterns are shown in Figure 3c (The codes of the meta‐atoms on the CAP metasurface for different radiated beam scanning angles are shown in Figure S5b, Supporting Information). The far‐field SLL of the metasurface using complex‐amplitude modulation is less than −15 dB, showing that the proposed complex‐amplitude modulation can not only suppress higher harmonic *β*_0,3, ‐_, but also further reduce the SLL in the far‐field patterns.

The CAP metasurface prototype is fabricated for verification of the design. The manufactured sample and testing environment are provided in Text S5 (Supporting Information). To confirm that reducing the meta‐atom spacing can suppress higher harmonic *β*_−1,3,+_, the far‐field control capability of this design is simulated and experimentally verified using phase‐only modulation, and the results are shown in Figure 3d,e. The simulated and measured far‐field SLLs at 5.4 GHz are less than −10 dB at beam scanning angles of −45° to +45° by step of 15°. For a beam scanning angle of 45°, the simulated and measured far‐field patterns show sidelobe levels at *θ* = −35° and *θ* = −31° of −9 and −6 dB, respectively, which is caused by *β*_0,3,‐_. The difference between simulated and measured maximum SLL angles is caused by fabrication tolerance, which affects the *k*_s_ of the SIW structure. The simulated and measured realized gains with different beam scanning angles are shown in Figure 3f. The maximum realized gain in simulation and measurement within the scanning range of −45° to 45° is 12.2 dBi and 10.9 dBi, respectively, while the minimum values are 10.4 dBi and 8.6 dBi, respectively. The maximum and minimum simulated radiation efficiency at 5.4 GHz within the beam scanning range of −45° to 45° is 59% and 48%, respectively, as provided in Text S6 (Supporting Information). From Text S6 (Supporting Information), it can also be seen that good beam scanning performance can be achieved from −45 ° to 45 ° in the range of 5.0 to 6.0 GHz. In the range of 5.0 to 6.0 GHz, the achieved gain reaches its peak at the center frequency ≈5.6 GHz and decreases when the operating frequency is far away from the center frequency.

To verify the suppression effect of the complex‐amplitude modulation on the far‐field SLL, simulations, and experiments are carried out at 5.4 GHz for the same range of beam scanning angles, as shown by the results in Figure 3g–i. The simulated maximum SLLs are found to be −14.5, −15.4, −16.8, −16.0, −18.7, −18.5, and −15.2 dB, respectively. Correspondingly, the measured maximum SLLs are −15.2, −14.3, −17.0, −14.0, −14.8, −16.2, and −14.3 dB. It can be concluded from Figure 3g,h that complex‐amplitude modulation not only suppresses the sidelobe generated by higher‐order harmonic *β*_0,3,‐_ effectively but also reduces the SLL at other angles. The maximum simulated and measured realized gain is 9.4 and 7.7 dBi, respectively, while the minimum values are 7.5 and 4.7 dBi, respectively. The maximum and minimum simulated radiation efficiency for complex‐amplitude modulation at 5.4 GHz within the beam scanning range of −45° to 45° are 44% and 35%, respectively, as presented in Text S6 (Supporting Information). The realized gain is lower than that of 1‐bit phase modulation which is caused by the implementation of complex‐amplitude modulation. When some meta‐atoms are turned off, the energy radiated into space will be lower compared to 1‐bit phase modulation. The realized gains can be further improved by increasing the radiation efficiency of a single meta‐atom or the number of meta‐atoms. It is worth noting that the low SLL beam scanning characteristics mentioned above constitute only an example of complex amplitude modulation for high‐quality control of far‐field beams. The CAP metasurface designed in this work has also the potential to achieve more forms of customized wavefronts.

### 3.2 Airy Beam Generation

The excellent far‐field control capability of the proposed meta‐atom has been demonstrated in the previous section, and its ability to synthesize Airy beams is also verified here. Compared to other nondiffracting beams, Airy beams possess self‐bending characteristics, providing higher degrees of freedom in wireless energy transmission and near‐field wireless communications. Although dynamic generation of Airy beams was achieved through phase modulation,^[^ ^28^, ^75^ ^]^ complex amplitude modulation is necessary to further improve the quality and provide more freedom for Airy beam generation.^[^ ^29^, ^76^ ^]^ In addition, by superposing an additional linear gradient phase profile to the Airy beam phase profile, the direction of the main lobe can be flexibly manipulated. The initial field envelope of the 1‐D Airy beam can be expressed as

(9) $$\phi \left(x\right)=Ai\left(bx\right)\exp\left(ax+jxk_{0}\sin\theta_{0}\right)$$

where *a* is a positive value, *b* is the transverse scale, and *θ*_0_ is the additional bending angle of the beam. For *Ai*(*x*) > 0, the phase modulation profile is *φ*_obj_ = arg(ϕ(*x*)) = *xk*_0_sin*θ*_0_ and for *Ai*(*x*) < 0, *φ*_obj_ = arg(ϕ(*x*)) = *π*+ *xk*_0_sin*θ*_0_.

The initial phase *φ_ini_* generated by waveguide feeding can be expressed as

(10) $$\varphi_{\mathrm{ini}}=-k_{s}x$$

Therefore, the phase shift *φ_Airy_* required to excite the Airy beam is

(11) $$\varphi_{\mathrm{Airy}}=\varphi_{\mathrm{obj}}\varphi_{\mathrm{ini}}^{*}$$

To achieve complex‐amplitude modulation, the quantized amplitude *A*_qua_airy_(*x*) of the meta‐atom is expressed as

(12) $$A_{\mathrm{qua}\_\mathrm{Airy}}\left(x\right)=\left\{\begin{array}{cc} radiating & ,abs(Ai(x))>t\\ non-radiating & ,abs(Ai(x))<t \end{array}\right.$$

where *t* is the threshold of the amplitude, which is schematically shown in Figure S10a (Supporting Information). More details of the Airy beam with the complex amplitude modulation are provided in Text S7 (Supporting Information). It can be observed that compared to only‐phase modulation, the Airy beam generated by complex‐ amplitude modulation achieves different lobes attenuation trends that are closer to the theoretical value.

**Figure 4:**
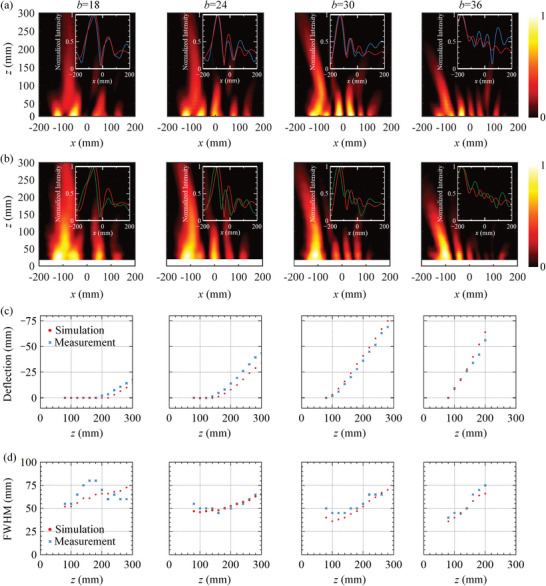
Airy beams with different scale levels *b* = 18, *b* = 24, *b* = 30 and *b* = 36. a) Simulated electric field distribution in the *xoz* plane, where the inset shows the simulated (blue line) and theoretical (red line) normalized intensity at *z* = 200 mm. b) Measured electric field distribution in the *xoz* plane, where the inset shows the measured (green line) and theoretical (red line) normalized intensity at *z* = 200 mm. c) Deflection of the beam along *z* direction extracted from simulations and measurements. d) FWHM of the beam extracted from simulations and measurements.

To analyze the influence of the transverse coordinate on the parabolic trajectory of the Airy beam, four Airy beams with different scale levels of *b* = 18, *b* = 24, *b* = 30, and *b* = 36, are designed, simulated, and experimentally verified. The amplitude of the Airy function is designed using Equation (11), where *t* is set as 0.3. The simulated and measured results of the electric field distribution at 5.4 GHz are shown in **Figure** **4**a,b, respectively. It can be observed that high‐quality Airy beams are generated by applying different bias voltages. The insets in Figure 4a,b show the Airy beam curve extracted at z = 200 mm. Among them, the red, blue, and green curves represent theoretical, simulated, and measured results, respectively, where the simulated and measured curves are well consistent with the theoretical ones. The simulated radiation efficiency of Airy beams for *b* values of 18, 24, 30, and 36 are 45%, 43%, 45%, and 38%, respectively, as given in Text S8 (Supporting Information). The observed small deviation is caused by the discretization loss and fabrication tolerance. In addition, to further verify the self‐bending characteristics of the Airy beams, their deflection trajectories are calculated, as shown in Figure 4c, which is defined as the difference between the center position of the Airy profile and the propagating hypothetical beam. It can be observed that both the simulated and measured results show a smooth parabolic trajectory. To evaluate the nondiffraction characteristics of Airy beams, the full width at half maximum (FWHW) of the Airy beam is also calculated, as shown in Figure 4d. The comparison between FWHM values obtained from measurements and simulations shows a good agreement. The minimum and maximum values of measured FWHW for *b* = 18, *b* = 24, *b* = 30, and *b* = 36 are 55, 55, 50, and 40 mm, respectively, and 80, 65, 65, and 75 mm, respectively. Except for *b* = 36, the maximum FWHW is less than 1.5 times the minimum FWHW within the range of *z* lower than 300 mm. This indicates that good nondiffraction characteristics are exhibited in the range of *z* lower than 300 mm. However, for *b* = 36, the beam undergoes diffraction due to a larger bending angle and a relatively smaller aperture length. Consequently, selecting a transformer scale is crucial when generating Airy beams to ensure their nondiffractive propagation characteristics.

**Figure 5:**
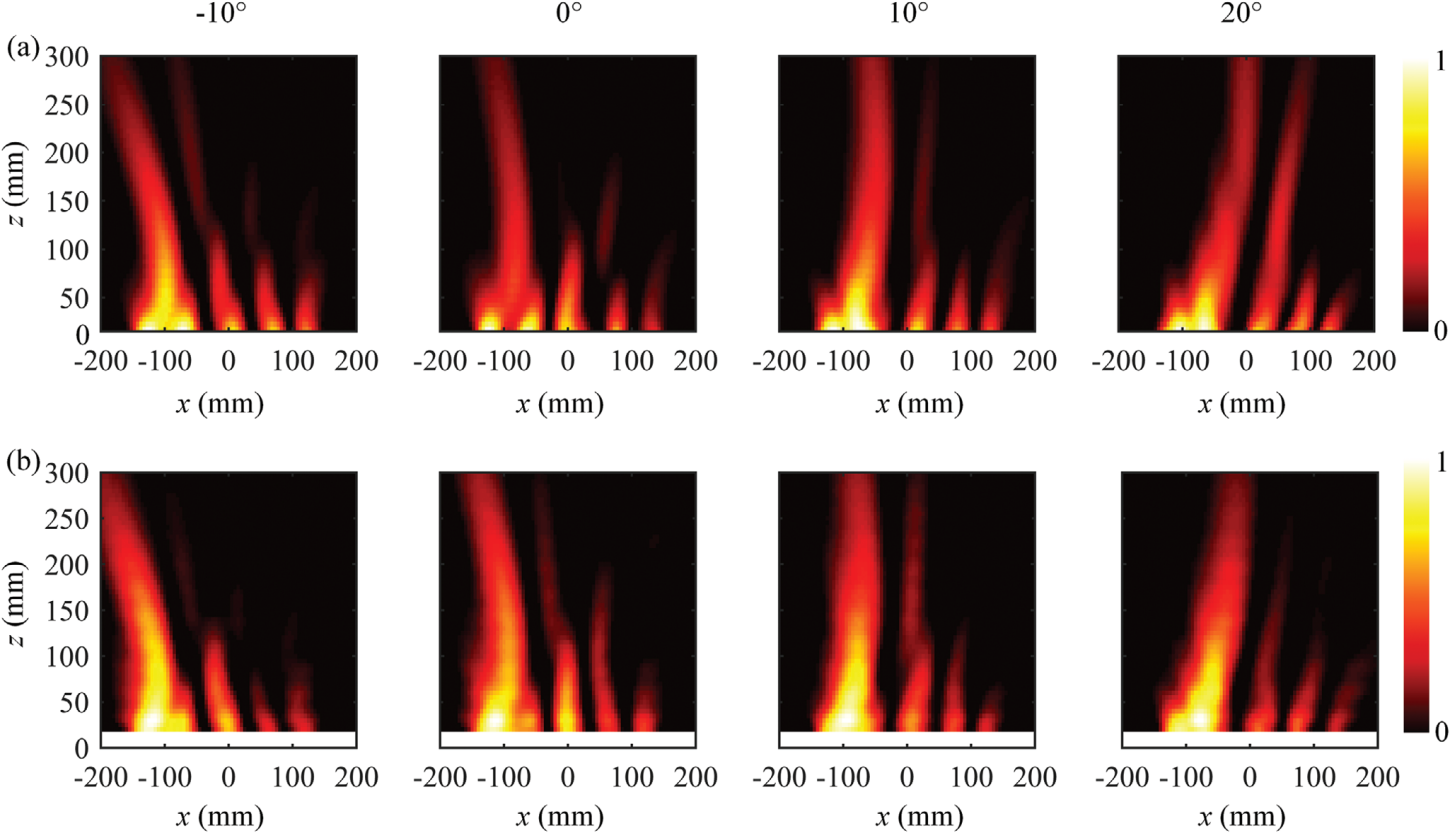
Beam steering of the Airy‐beam with *b* = 24 at 5.4 GHz. a) Simulated electric field distribution in the *xoz* plane for steering angles of −10°, 0°, 10°, and 20°. b) Measured electric field distribution in the *xoz* plane for steering angles of −10°, 0°, 10°, and 20°.

To further demonstrate the flexibility of the CAP metasurface for Airy beam manipulation, the beam steering characteristics are studied. According to Fourier transforms theory, the superposition of a linear gradient phase profile leads to a bending effect on the original wave, as shown in Equation (9). Four different bending angles (−10°, 0°, 10°, and 20°) for the configuration with Airy function parameters *b* = 24 are tested. As presented by the results in **Figure** **5**, the simulated and measured results have good consistency, indicating the achievement of flexible control on the propagation direction of the Airy beam. The dynamic control of the propagation direction provides more application potential for wireless energy transfer, communications, and sensing.

## 4 Conclusion

In conclusion, we demonstrated a guided wave‐driven metasurface that can achieve dynamic and simultaneous control of amplitude and phase. The metasurface is fed by a traveling wave in an SIW structure, which solves the integration challenge of traditional metasurface fed by external spatial sources and avoids a complex power divider network of radiation‐type metasurfaces. 1‐bit amplitude switching between radiating and nonradiating states and 1‐bit phase switching between 0° and 180° radiation phase can be achieved by controlling the states of the p‐i‐n diodes loaded in each meta‐atom. The proposed metasurface can manipulate advanced EM functions of low SLL far‐field beam scanning and high‐quality Airy beam generation. Combined with GA, the proposed metasurface can achieve beam scanning from −45° to 45° with an SLL of less than −14 dB. In addition, the high‐quality Airy beam generator demonstrates that our scheme can also be widely applied in near‐field EM wave manipulation. Experimental results show good agreement with simulated and theoretical ones, verifying the excellent performance of the proposed metasurface. Such complex‐amplitude, programable, and guided wave‐driven metasurface can find great application potential in microwave communications, sensing, and wireless energy transfer.

## 5 Experimental Section

### Sample Fabrication

The designed guided‐wave‐driven metasurface was manufactured using standard multi‐layer printed circuit board (PCB) technology. The 122 Skyworks SMP1321‐079LF p‐i‐n diodes were integrated onto the guided‐wave driven metasurface by machine welding. The total surface area of the fabricated sample was 420 × 60 mm^2^ and the total thickness was 3.15 mm (≈0.05 *λ*_0_).

### Simulation

The time‐domain solver of the commercial CST Studio Suite simulation software package was used to numerically simulate the *S*‐parameters, radiated energy, radiation phase, and surface current of the elementary meta‐atom. To verify the control ability of the metasurface in near‐field and far‐field regions, a simulation model was designed as shown in Figure S1 (Supporting Information), which uses the same dielectric material and size structure as the fabricated sample. In the numerical simulation process, the equivalent circuits of the p‐i‐n diode shown in Figure 2d were used to simulate the switching characteristics of the p‐i‐n diode.

### Experimental Setup

To avoid the influence of the testing environment on the measured results, the far‐field and near‐field characteristics of the metasurface are tested in a microwave anechoic chamber, as shown in Figure S6d,e (Supporting Information). A Keysight P9384B vector network analyzer (VNA) was used to measure and record the far‐field patterns and electric near‐field distributions. The realized gain of the metasurface was measured using a comparison method, with the standard gain XB‐HA159‐15 horn antenna operating from 4.64 to 7.05 GHz.^[^ ^77^ ^]^

## Conflict of Interest

The authors declare no conflict of interest.

## Supporting information

Supporting Information

## Data Availability

The data that support the findings of this study are available from the corresponding author upon reasonable request.
