# Supplementary material for: Complex‐Amplitude Programmable Versatile Metasurface Platform Driven by Guided Wave
Source: Adv Sci (Weinh). 2024 Mar 14;11(19):2309873. doi: 10.1002/advs.202309873 (PMC11109637; doi:10.1002/advs.202309873)
Supplement: Supplementary file 1 — Supporting Information [file ADVS-11-2309873-s001.pdf]

ADVANCED SCIENCE
Open Access

Supporting Information

for *Adv. Sci.*, DOI 10.1002/advs.202309873

Complex-Amplitude Programmable Versatile Metasurface Platform Driven by Guided Wave

Jian-Qiao Han, Fan-Yi Meng, Chunsheng Guan*, Cong Wang, Tao Jin, Tong Cai, Chang Ding*, Shah Nawaz Burokur, Qun Wu and Xumin Ding**

Complex-Amplitude Programmable Versatile Metasurface Platform Driven by Guided Wave

Jian-Qiao Han, Fan-Yi Meng, Chunsheng Guan*, Cong Wang, Tao Jin, Tong Cai, Chang Ding*, Shah Nawaz Burokur, Qun Wu and Xumin Ding**

Text S1. Overall Schematics of the metasurface

Text S2. Impact of DC bias network on meta-atom characteristics

Text S3. Principle of far-field beam scanning

Text S4. Design details of the genetic algorithm (GA)

Text S5. Metasurface prototype fabrication and experimental setups

Text S6. Working bandwidth and radiation efficiency for far-field beam scanning

Text S7. Airy beam generation

Text S8. Radiation efficiency for Airy beam generation

Text S1. Overall schematics of the metasurface

The schematics of the metasurface profile, the DC bias network, and the PIN pads are shown in **Figues S1a-c**, respectively. The DC bias network marked in orange is distributed on the top and bottom faces of Dielectric 3, and connected with metal blind vias.

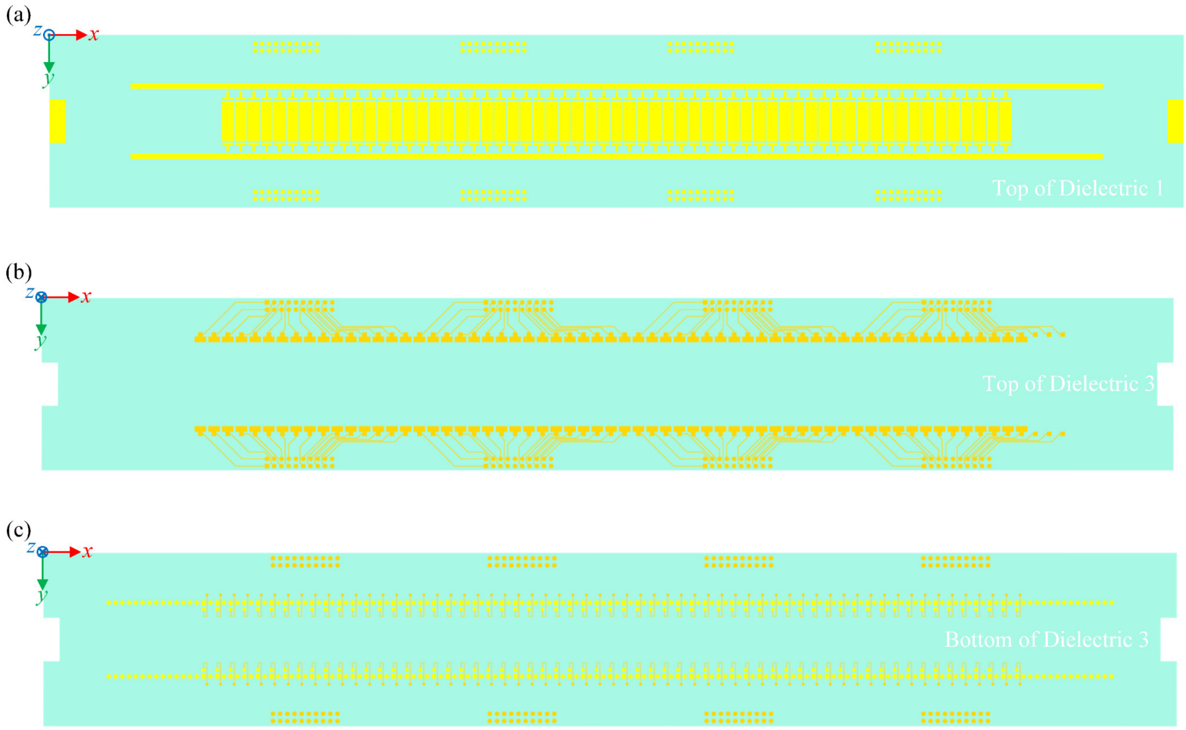

Figure S1. Schematic diagram of guided wave-driven metasurface. a) Top layer. b) DC bias network. c) PIN pads.

Text S2. Impact of DC bias network on meta-atom characteristics

To avoid RF power leakage into the DC bias network, a microstrip line with a length of 10.8 mm and a width of 0.2 mm is used to connect the positive terminal of the p-i-n diodes at one end, and the other end is connected to a rectangular metal patch of 4 mm × 2 mm located on the upper face of Dielectric 3 through a blind via.

To analyze the impact of DC bias network on the element characteristics, the *S* parameters are simulated for both bias and unbiased meta-atoms, and the results are shown in **Figure S2**. It can be found that the *S* parameters are consistent, verifying that the designed bias network has very little influence on the characteristics of the meta-atom.

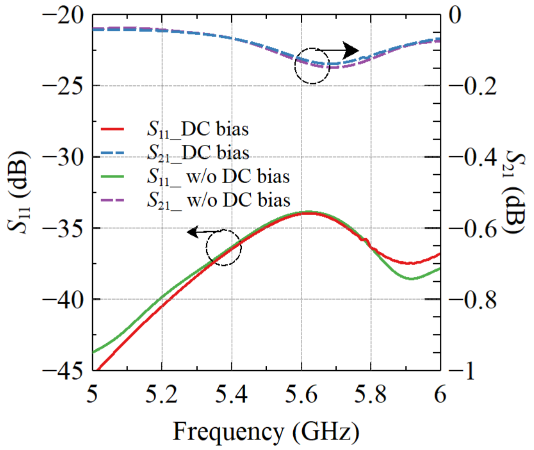

Figure S2. Simulated *S*-parameters with and without DC bias.

Text S3. Principle of far-field beam scanning

When the designed guided-wave driven metasurface operates in far-field, its working principle is shown in **Figure S3**. Compared with traditional space-fed metasurfaces, the phase shift provided by guided-wave driven metasurfaces can be divided into two parts: (1) The phase delay provided by transmission lines; (2) Phase response of each meta-atom. Among them, the phase delay provided by the transmission line to each meta-atom can be expressed as

$$\varphi_{\text{ref}} = -k_x(n-1)d \tag{S1}$$

When the angle of the radiated beam is θ_0, the wavefront phase distribution of the metasurface can be expressed as

$$\varphi_{obj} = -k_0 \sin\theta_0 (n-1)d \tag{S2}$$

Therefore, the phase response for each meta-atom φ_n is given as

$$\varphi_n = \varphi_{obj} - \varphi_{ref} + \varphi_0 = n \times d \times \left(k_s - k_0 \sin\theta_0\right) + \varphi_0 \tag{S3}$$

Among them, φ_0 is the initial phase.

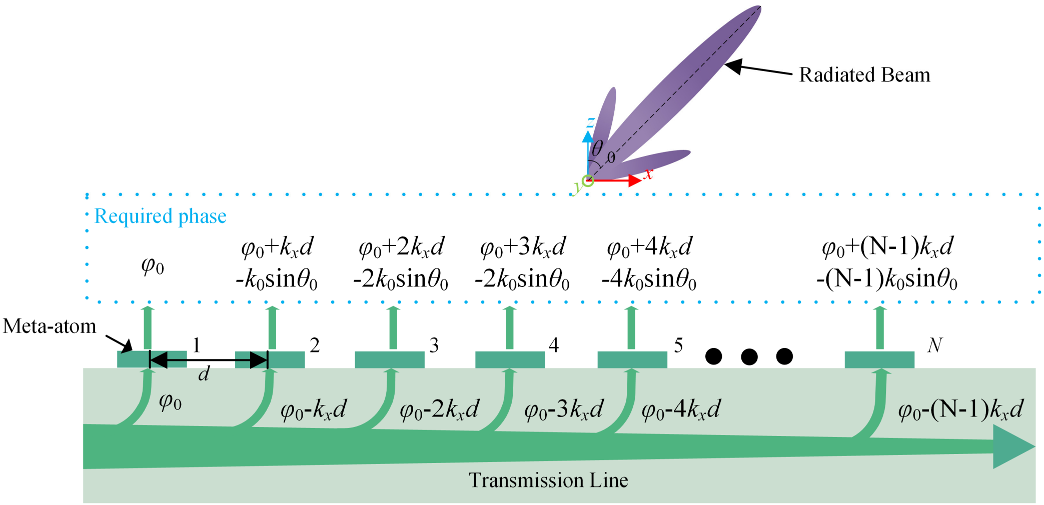

Figure S3. Working principle for far-field radiation of the guided wave-driven metasurfaces.

Text S4. Design details of the genetic algorithm (GA)

Although higher harmonics radiation caused by 1-bit phase modulation can be effectively suppressed by reducing the distance between elements, there is a limit for the reduction of element spacing. In this study, the designed meta-atom not only has the function of 1-bit phase regulation, but also controls the radiating state of the meta-atom. Therefore, GA is used to optimize the amplitude distribution of the metasurface, thereby breaking the periodicity of 1-bit phase modulation, further suppressing higher order mode radiation, and reducing far-field sidelobe levels (SLLs). The metasurface designed in this study is a one-dimensional array composed of N elements (N = 61) and the amplitude of each element can be represented by the numbers 0 and 1, which correspond to the non-radiating and radiating states of the elements, respectively. Although the total number of radiating meta-atoms can be guaranteed to remain unchanged during the use of GA optimization, the non-radiating state of the first and last meta-atom will change the aperture length of the array, leading to a decrease in array directivity. Therefore, in order to maintain the same metasurface aperture, the distribution of the array needs to meet the following conditions

$$\begin{cases} A(1)=1 \\ A(N)=1 \\ \sum_{n=1}^{N} A(n)=NL \end{cases} \tag{S4}$$

where $A(1)$ represents the state of the first meta-atom, $A(N)$ represents the state of the last meta-atom, $A(n)$ represents the state of the n-th meta-atom, and NL represents the number of meta-atoms in radiation state. The maximum sidelobe level $MSLL$ of a unit can be expressed as

$$MSLL = \max_{\phi \in S}\left[F(\theta)\right]$$
$$S = \left\{\theta \mid -90^\circ \le \theta \le \theta_0 - \varphi_0 \cup \theta_0 + \varphi_0 \le \theta \le 90^\circ\right\} \tag{S5}$$

where, θ_0 is the radiated beam pointing angle, S is the sidelobe interval, φ_0 is the spacing angle from the zero power point of the radiated beam to the maximum power point. Therefore, the optimization model can be expressed as

$$fitness = \min_{A}(MSLL) \tag{S6}$$

By optimizing the value of A, the amplitude distribution of the metasurface is optimized to obtain low SLLs. The flowchart for optimizing the far-field side lobe level using a genetic algorithm is shown in **Figure S4**. In this optimization process, the maximum iteration count I_{MAX} is employed as the criterion to determine the termination of the optimization process, with the output of the optimal individual upon completion of the iteration process. The optimization process involves the following steps:

1) Determine the number of individuals in the population *NP* (*NP*=500), each with a dimension *N* (equal to the number of meta-atoms in the metasurface), and an initial radiated beam pointing angle θ_0;
2) Initializing the population, ensuring that each individual satisfies the optimization condition as indicated by Equation (S4);
3) Start the iteration process.

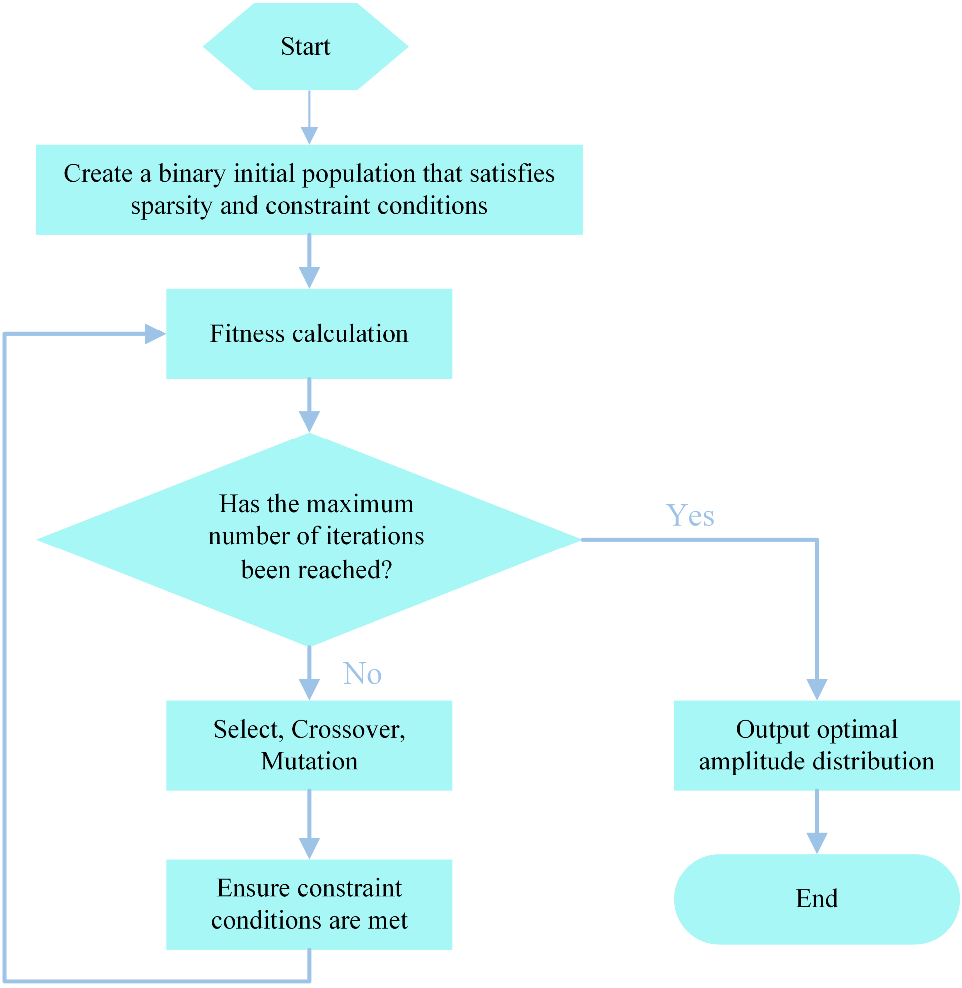

Figure S4. Flowchart of the GA procedure.

The results obtained by optimizing the SLLs of different radiation unit numbers *NL* (30, 35, 40, 45, 50, 55) within the scanning range of -45 ° to 45 ° are shown in **Table S1**. It is worth noting that when *NL* is 40, the optimization result of the SLL is superior to the others. Therefore, *NL* of 40 is selected for subsequent simulations and experiments. **Figure S5** shows the coding sequences at different angles of phase-only modulation and complex-amplitude modulation.

Tabel S1. Maximum SLL after optimization with different number of radiating meta-atoms.

		Number of radiation meta-atoms (*NL*)					
		30	35	40	45	50	55
Radiated beam pointing angles θ_0 (°)	-45	-16.0	-16.6	-17.1	-18.0	-17.9	-16.1
	-30	-15.5	-15.8	-16.7	-16.4	-16.2	-16.0
	-15	-15.7	-16.7	-17.1	-17.4	-17.7	-17.0
	0	-17.0	-16.2	-17.5	-17.7	-17.0	-15.0
	15	-16.0	-16.8	-18.0	-17.5	-16.8	-14.8
	30	-17.0	-19.0	-18.4	-17.4	-17.6	-16.4
	45	-16.8	-16.8	-17.5	-16.0	-14.5	-12.8

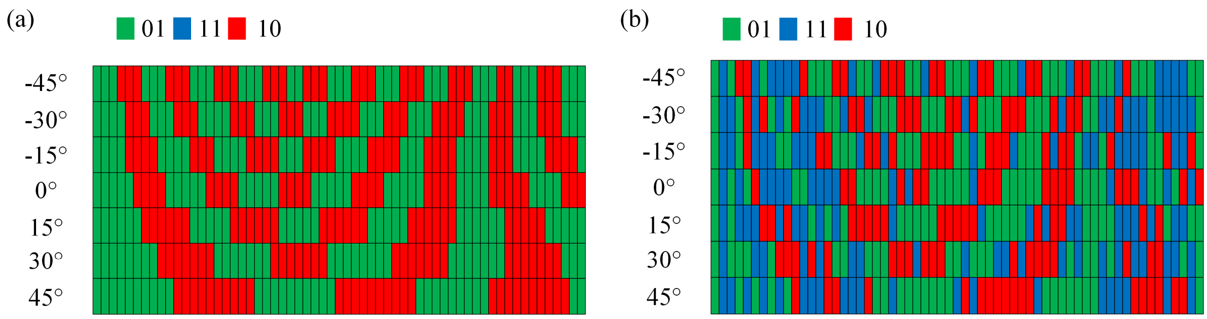

Figure S5. a) Coding sequences for different scanning angles with phase-only modulations. b) Coding sequences for different scanning angles with complex-amplitude modulation.

Text S5. Metasurface prototype fabrication and experimental setups

The guided wave-driven metasurface prototype is fabricated for proof-of-concept validation. The fabricated prototype is shown in **Figures S6a,b**. The size of the CAP metasurface prototype is 420 mm × 60 mm × 3.15 mm. The prototype is dynamically regulated using a logic control board as shown in **Figure S6c**. The output port of the logic control board can output two voltage values (0 V or 0.85 V) to control the state of the PIN diodes. The far-field and near-field measurement setups are shown in **Figures S6d,e**, respectively. Two SMA connectors are welded to both ends of the SIW feed network, with one end connected to the vector network analyzer (VNA) via a coaxial cable and the other end connected to the matching load. During far-field measurement, a standard horn antenna is used as the receiving antenna to measure the far-field patterns. In near-field tests, a probe is used as the receiving end.

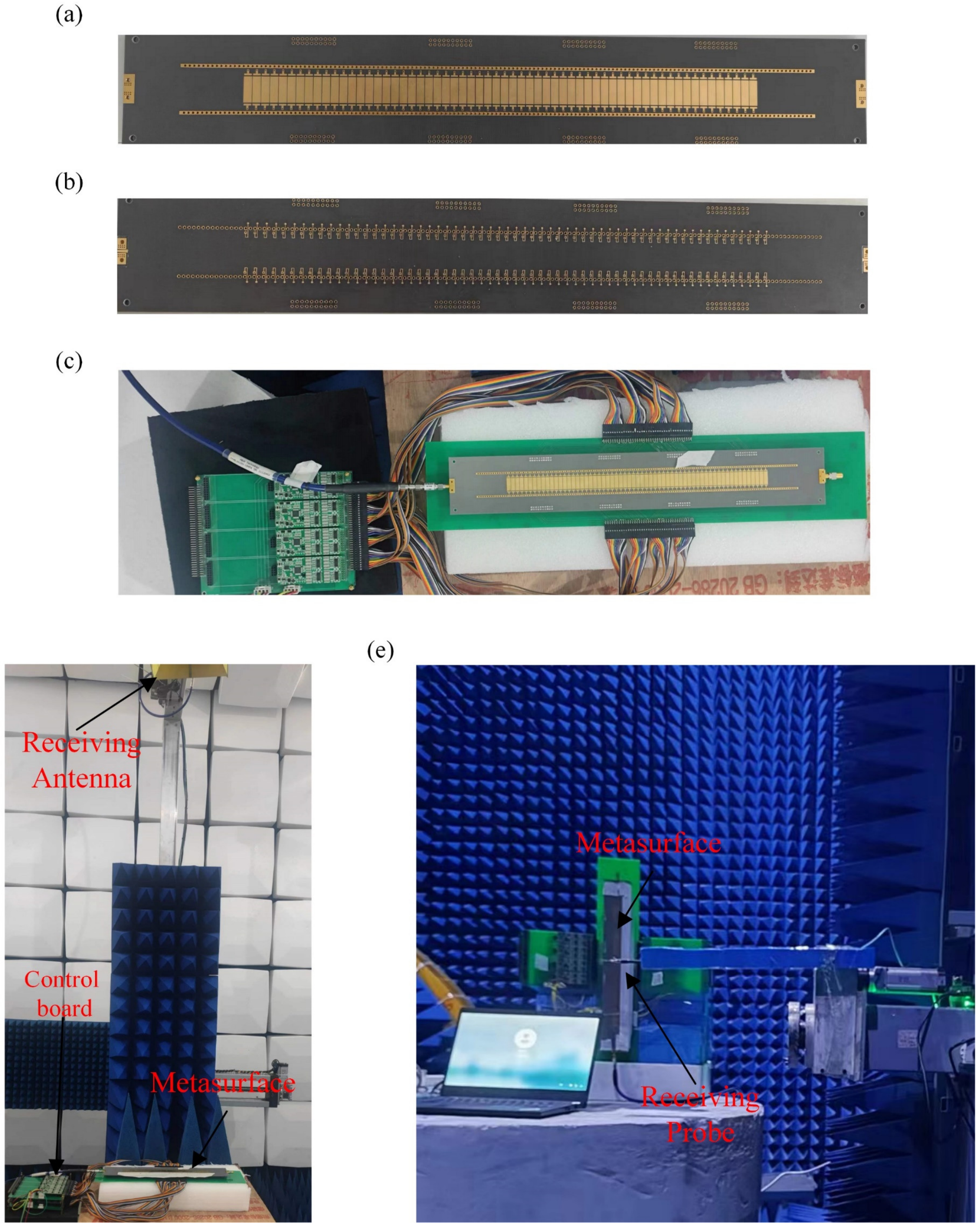

Figure S6. Metasurface prototype and experimental setups. a) Top view of the fabricated metasurface. b) Bottom view of the fabricated metasurface. c) Schematic diagram of the metasurface connected with the logic control board. d) Far-field experimental setup. e) Near-field experimental setup.

Text S6. Working bandwidth and radiation efficiency for far-field beam scanning

To further verify the performance within the working bandwidth, we conducted simulation analysis on the beam scanning at angles of -45°, 0°, and 45° in the frequency range of 5.0 to 6.0 GHz, with a frequency step of 0.2 GHz. From **Figure S7**, which shows the far-field results, it can be observed that the expected beam pointing angle is achieved in the frequency range of 5.0 to 6.0 GHz. S_{11} and S_{21} results are shown in **Figure S8a,b**, respectively. It

can be observed that in the 5.0 to 6.0 GHz range, the metasurface exhibits good impedance matching characteristics. **Figure S8c** shows the simulated realized gain versus frequency and it can be observed that the realized gain reaches a peak value at the central frequency of 5.6 GHz, and decreases when we move away from the central frequency. The realized gains at 5.0 GHz for beam pointing angles of -45 °, 0 °, and 45 ° are 6.78, 7.65, and 6.19 dBi, respectively. The realized gains at 6.0 GHz are 7.16, 7.23, and 6.48, respectively. Although the realized gains are lower at 5.0 and 6.0 GHz, as shown in **Figure S8c**, the transmission coefficient fluctuates in the range of -3.5 to -5.0 dB, indicating that a significant amount of electromagnetic energy has not been radiated. Therefore, the realized gain of the metasurface can be improved by increasing the number of meta-atoms.

Figure S9a,b shows the simulation results of the power budget for different beam scanning angles at 5.4 GHz for phase-only and complex-amplitude modulation scenarios, respectively. It can be observed that the power loss in the metasurface remains almost similar for both scenarios. However, when performing complex-amplitude modulation, the radiated energy is smaller than when only-phase modulation is performed. The simulated radiation efficiency for the different beam scanning angles at 5.4 GHz are shown in **Figure S9c**, in which the maximum radiation efficiency values for phase-only modulation and complex-amplitude modulation within the beam scanning range of -45° to 45° are 59% and 44%, and the minimum values are 48% and 35%, respectively.

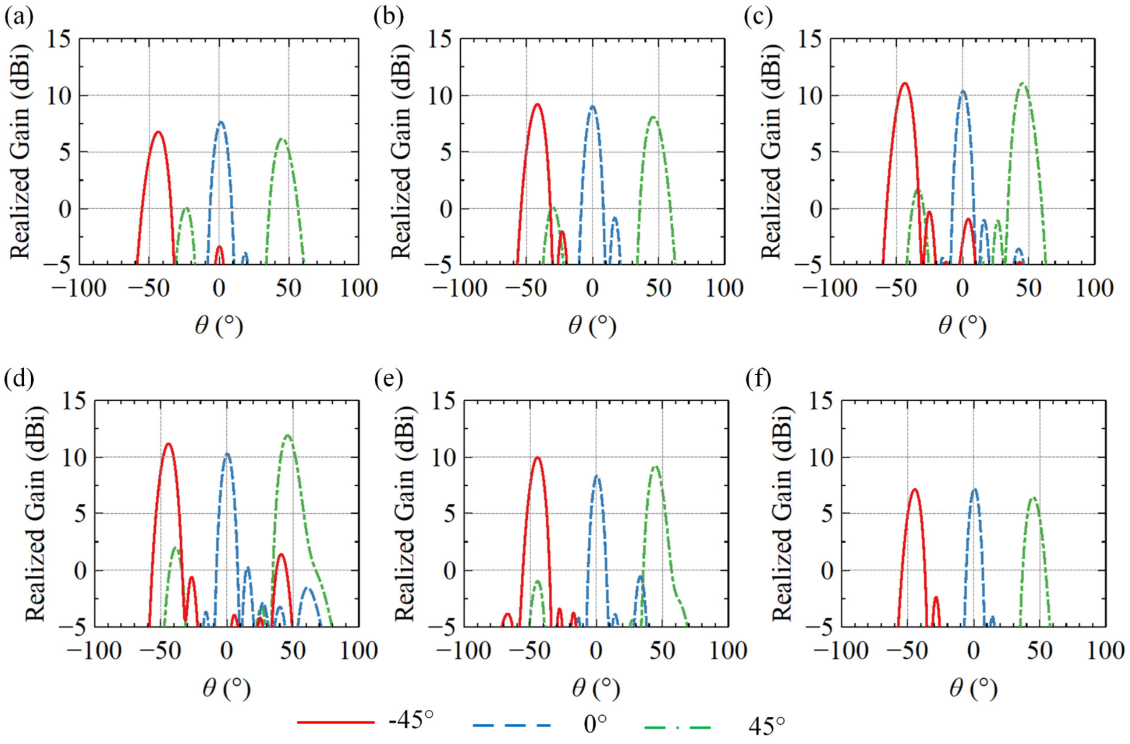

Figure S7. Simulated far-field radiation patterns at different frequencies for beam scanning angles of -45°, 0°, and 45°. a) 5.0 GHz. b) 5.2 GHz. c) 5.4 GHz. d) 5.6 GHz. e) 5.8 GHz. f) 6.0 GHz.

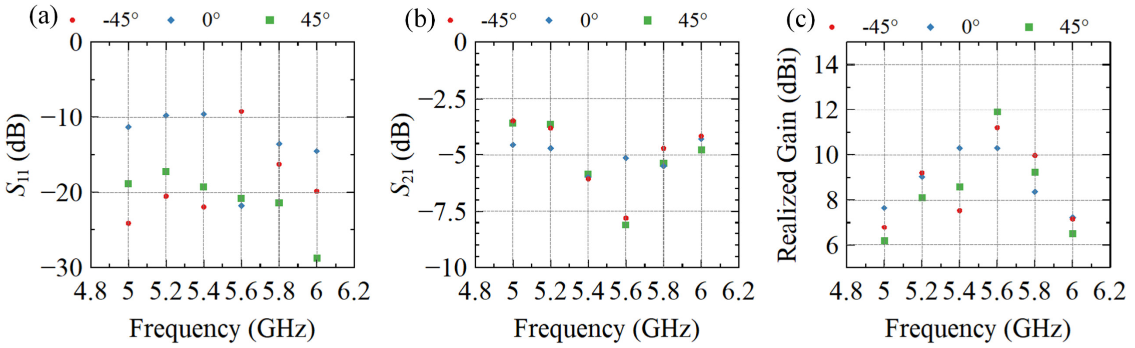

Figure S8. Simulated *S*-parameters and realized gain versus frequency. a) S_{11} (dB). b) S_{21} (dB). c) Realized gain (dBi).

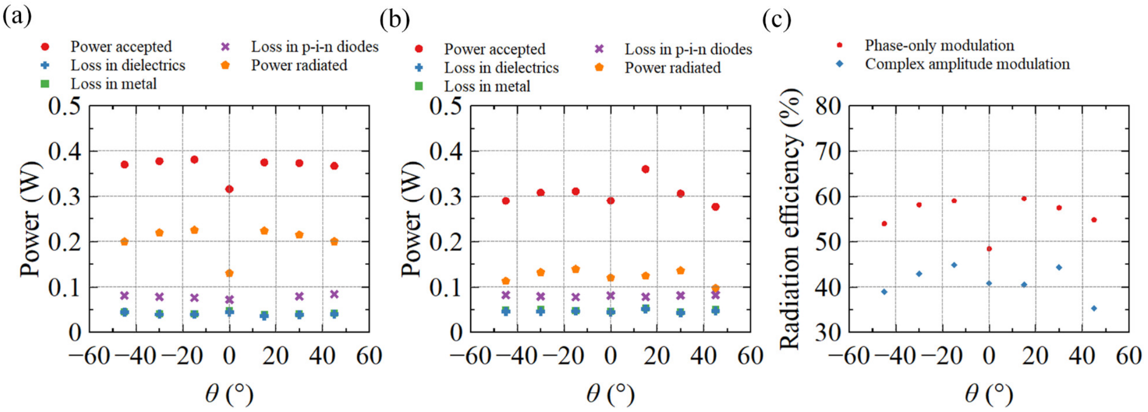

Figure S9. Simulation results of the guided-wave driven metasurface with different beam scanning angles at 5.4 GHz. a) Power budget for phase-only modulation. b) Power budget for complex-amplitude modulation. c) Radiation efficiency.

Text S7. Airy beam generation

In order to study the influence of the threshold t in Equation (12) of the main text, the electric field distribution at $t = 0$, $t = 0.30$, and $t = 0.39$ are simulated, respectively. **Figure S10b** gives the state of the meta-atom for different values of t with $a = 0.60$, $b = 30$. **Figures S10c,e** show the simulation results at 5.4 GHz. The insets in Figure **S10c,e** show the comparison of the normalized intensities between the simulated (blue line) and theoretical results (red line) at 200 mm from the metasurface. **Figure S10c** shows the Airy beam with phase-only modulation ($t = 0$), and although the self-bending and nondiffracting features can be observed, a large difference between the distribution of the simulated normalized intensity in the near-field and that of the ideal Airy beam can be noticed from the inset of Figure **S10c**. Figures **S10d,e** show the Airy beams for the cases $t = 0.30$ and $t = 0.39$, respectively, which show better consistency with the theoretical results. Thus,it can be concluded that complex-amplitude modulation can achieve better Airy beam synthesis.

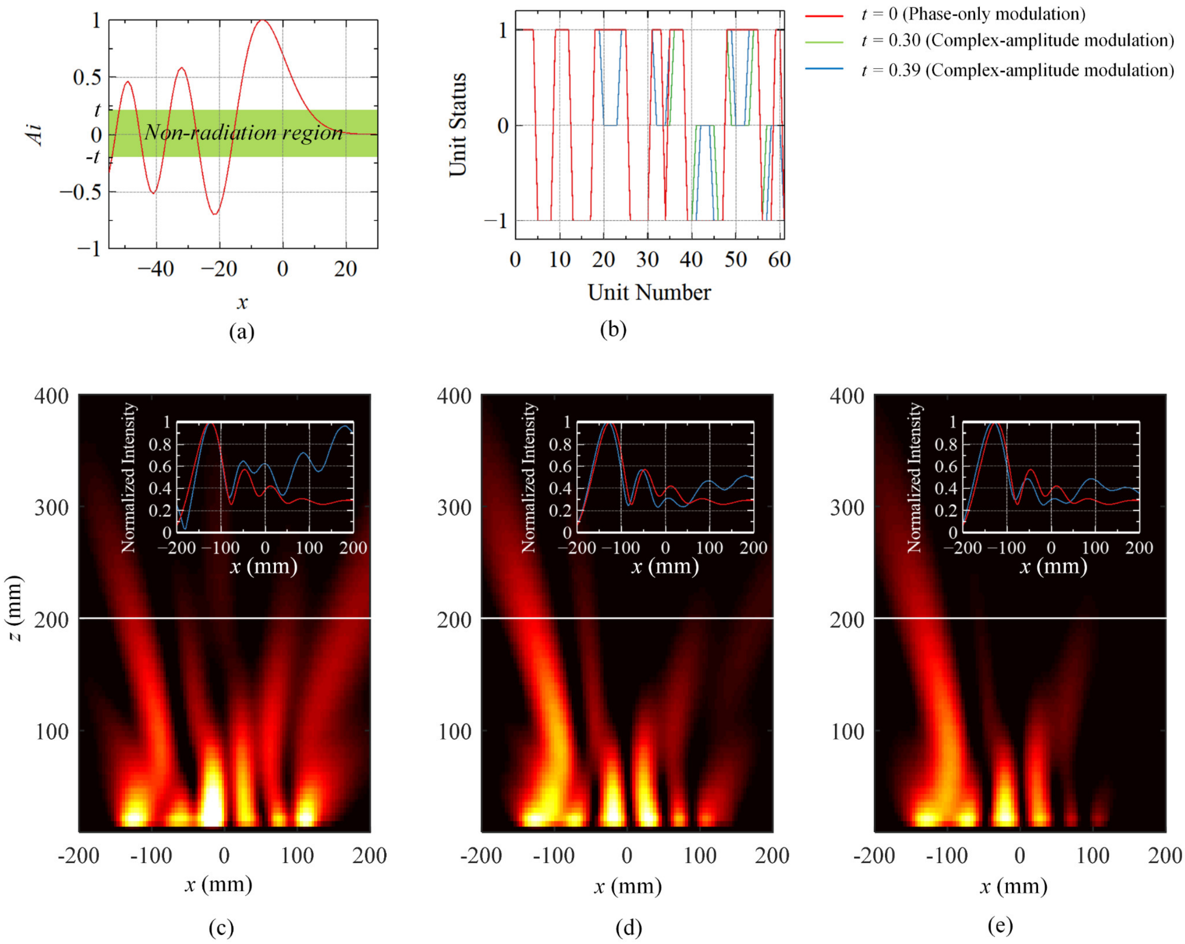

Figure S10. a) Normalized Airy function. b) Meta-atom states at different values of t. c) Airy beam simulation results with phase-only modulation. d) Airy beam simulation results with complex-amplitude modulation ($t = 0.30$). e) Airy beam simulation results with complex-amplitude modulation ($t = 0.39$). The insets compare the normalized intensity between the simulated (blue line) and theoretical results (red line).

Text S8. Radiation efficiency for Airy beam generation

The simulated power budget and radiation efficiency of the Airy beams for b values of 18, 24, 30, and 36 are shown in **Table S3**, and the simulated radiation efficiency of the Airy beams are 45%, 43%, 45%, and 38%, respectively.

Table S3. Simulated results of power budget and radiation efficiency for Airy beams with the value of *b* is 18, 24, 30, and 36.

b	Power loss in dielectric (W)	Power loss in metal (W)	Power loss in diodes (W)	Power radiated (W)	Power accepted (W)	Radiation efficiency (%)
18	0.044	0.045	0.072	0.129	0.290	45
24	0.047	0.048	0.073	0.125	0.293	43
30	0.043	0.045	0.074	0.132	0.294	45
36	0.045	0.048	0.075	0.104	0.272	38
